# Case Report: Intra-Tumoral Vaccinations of Quadrivalent HPV-L1 Peptide Vaccine With Topical TLR-7 Agonist Following Recurrence: Complete Resolution of HPV-HR-Associated Gynecologic Squamous Cell Carcinomas in Two Patients

**DOI:** 10.3389/pore.2021.1609922

**Published:** 2021-12-20

**Authors:** Mark Reedy, Shirisha Jonnalagadda, Komaraiah Palle

**Affiliations:** ^1^ Department of Obstetrics and Gynecology, School of Medicine, Texas Tech University Health Sciences Center, Lubbock, TX, United States; ^2^ Department of Cell Biology and Biochemistry, Texas Tech University Health Sciences Center, Lubbock, TX, United States

**Keywords:** human papilloma virus high-risk variant, radiation refractory squamous cell carcinoma, quadrivalent HPV-L1 vaccine, intratumoral injection, toll-like receptor 7 agonist, immunotherapy

## Abstract

The human papilloma virus (HPV) high-risk variants (HPV-HR) such as HPV16 and HPV18 are responsible for most HPV related cancers, including anogenital and head and neck cancers. Here, we present two patients with HPV-HR-associated gynecological malignancies who, after failing radiation therapy, were treated with experimental salvage immunotherapy regimen resulting in complete, durable responses in both patients. Each patient was diagnosed with recurrent, radiation-refractory, HPV-HR positive, squamous cell carcinoma of the lower genital tract. Patient A was a 90-year-old, African American, with metastatic vulvar cancer to the right inguinal-femoral triangle and pulmonary metastases. Patient B was a 41-year-old, Caucasian, with a central-recurrence of cervix cancer. Each patient received at least two intratumoral quadrivalent HPV-L1 vaccine (Gardasil™) injections and daily topical TLR-7 agonist (imiquimod) to the tumor surface 2 weeks apart. This combination of intratumoral vaccinations and topical TLR-7 agonist produced unexpected complete resolution of disease in both patients. The importance of radiation therapy, despite being considered a treatment failure by current definitions, cannot be understated. Radiation therapy appears to have offered a therapeutic immune advantage by modifying the tumor microenvironment. This immune protocol has potential to help patients with advanced HPV-HR-related malignancies previously considered incurable.

## Introduction

The human papilloma viruses (HPV) are responsible for ∼30% of viral-induced cancers. There are at least fourteen known oncogenic or high-risk HPV (HPV-HR) types. Prophylactic HPV vaccines have proven effective in preventing benign and malignant manifestations of this virus in both men and women. Unfortunately, these vaccines require at least 10–20 years “lead-in” time before showing an epidemiological-effect in cancer prevention. Despite the proven successes of these vaccines, society has neither embraced the significance nor adequately utilized this medical advancement. In addition, immunotherapies to effectively treat HPV-HR cancers have been elusive. We present two patients with HPV-HR positive gynecological malignancies who recurred following radiation therapy. Both patients were desperate and facing dismal prognoses. We describe the “off-label” use of two FDA-approved immune drugs consisting of intratumoral vaccinations of quadrivalent HPV-L1 vaccine (Gardasil™) and daily application of topical imiquimod resulting complete resolution of radiation refractory disease in both patients.

## Case Report

These are the only two patients treated with this combination of intra-tumoral vaccinations of quadrivalent HPV vaccine and topical imiquimod after presenting with radiation refractory disease. Each female patient had personal and logistical issues limiting travel for inclusion into regional/national research studies. Both patients requested alternative treatment options that could be administered during their current treatment choices. Following Bioethics Committee review and approval, of each patient was consented and received the immune regimen of intratumoral injections of quadrivalent HPV-L1 vaccine (Gardasil™) and topical imiquimod (Table 1). Demographic information for patients A and B is presented in Table 2.

**TABLE 1:** Immunotherapy protocol for patient A and patient B.

Immunotherapy protocol	Details
Quadrivalent HPV-L1 Antigen Vaccine (Gardasil™)	Volume-0.5 ml
	L1 antigens: 120 mcg; HPV 16 = 20 mcg; HPV 18 = 40 mcg; HPV 6 = 20 mcg; and HPV 11 = 40 mcg
Adjuvant used	Aluminum hydroxyphosphate = 225 mcg
Vaccination cycle	Intratumoral injections followed by application of topical imiquimod for fourteen nights
Number of planned cycles	3, if needed
Quadrivalent vaccine used per cycle	0.25 ml, or 60 mcg, of original vaccine volume
Imiquimod concentration used	Patient A applied one 250 mcg dose of 5% imiquimod (3M™)
	Patient B applied a compounded 0.2% imiquimod vaginal suppository (compounded locally)
Treatment: cycle #1	Intratumoral vaccination of Patient A and B using 0.25 ml quadrivalent vaccine diluted with 2.75 ml saline (total volume 3 ml) injected evenly throughout entire recurrent tumor
	Day 1: Imiquimod therapy applied immediately after intratumoral injection again that night
	Day 2–14: Imiquimod applied topically to surface of tumor by patient before bed
Treatment Cycle #2: Day #15	Both patients received the same treatment as cycle #1. In addition, each patient received a subcutaneous injection in the right shoulder as a “booster.” This was a subcutaneous injection and not IM. Subcutaneous injection offers immune stimulation of Langerhan cells which are also present in skin and mucosal/submucosal areas in which these tumors arise. We considered this offered a “prime-boost” effect. This was the only vaccination given that was not intratumoral
	Patient B received cycle #2 8 days before planned exenterative surgery
Treatment Cycle #3	Patient A missed her appointment 2 weeks following her second cycle
	Eleven weeks after cycle #2, she received the last intratumoral injection into a 2 cm^3^ tumor as shown in Figure 2B
	Patient B received only 2 cycles

**TABLE 2:** Demographic comparison between patients A and B.

	Patient A	Patient B
Age	90 years	41 years
Ethnicity	African-American	Caucasian
HPV + malignancy site	Vulva	Cervix
Grade of tumor	Grade I, squamous cell carcinoma	Grade 2, squamous cell carcinoma
Stage at initial diagnosis	Stage 2 vulvar cancer	Stage 3B cervical cancer
Initial therapy	Radical hemi-vulvectomy with bilateral sentinal lymph node biopsies	Cisplatin-based chemo-radiation plus two cesium low-dose rate tandem and ovoids
Recurrent cancer interval and location	3 years to right groin and bilateral pulmonary metastasis	3 weeks after second cesium implant with 3–4 cm tumor on cervix
Initial treatment of recurrence	Palliative fractionated external radiation (120 cGy to right groin/day for 10 fractions = 1200 cGy)	Planned pelvic exenteration
Intratumoral therapy started	7 days after completing radiation	4 weeks after second cesium implant

### Patient A

Patient A was a 90-year-old African-American female with recurrent, HPV-HR positive, squamous cell carcinoma (grade 1) of the vulva with metastatic disease to the right inguinal-femoral triangle (11 × 11 cm tumor) and bilateral pulmonary metastasis. Previously, this patient presented with a 4 cm peri-clitoral, grade 1, squamous cell carcinoma treated by radical hemi-vulvectomy and bilateral inguinal sentinel node biopsies. The vulvectomy specimen demonstrated no lymph-vascular space invasion and the specimen’s margins were clear. Bilateral inguinal sentinel lymph nodes, consisting of two nodes on the left and three nodes from right, were negative for malignant cells. No further therapy was required, and the patient never returned for follow-up until presenting 3 years later with 11 × 11 cm squamous cell carcinoma of right groin causing a right lower extremity deep vein thrombosis. Chest x-ray revealed a “snow storm” appearance from bilateral pulmonary metastasis (Supplementary Figure S1). The patient elected to enter Hospice to help manage end-of-life care. Due to the extreme right groin and leg pain, palliative external beam radiation and low-molecular weight heparin was initiated. Radiation therapy was discontinued following 10 fractions (total dose 1200 cGy) due to increasing leg pain and tumor progression to 13 × 13 cm (20% increase). The patient requested enrollment into an immunotherapy study treating HPV-HR, intraepithelial neoplasia of the lower genital tract. Bioethics’ Committee convened and approved the compassionate use of the immune therapy. The patient received the first intratumoral vaccination of the quadrivalent vaccine 7 days after palliative radiation was discontinued (Table 1).

**FIGURE 1:**
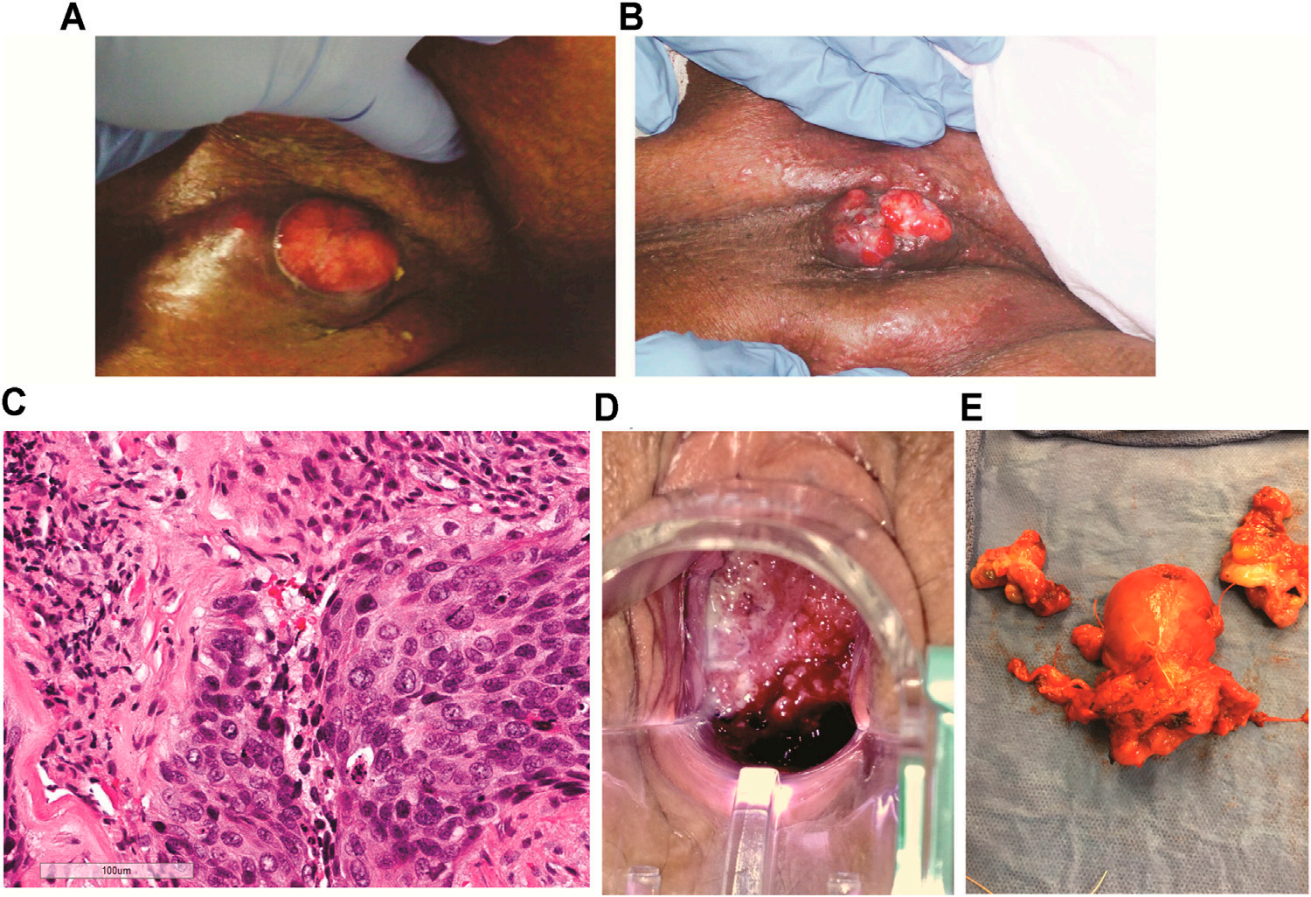
Tumor images of patients A and B. **(A)** Patient A, right inguinal squamous cell carcinoma 2 weeks after first cycle of experimental immune therapy. Tumor surface became smooth and epithelial margins symmetric compared to original tumor state. **(B)** 11 weeks after second experimental treatment, the patient returned for the last cycle of intratumoral vaccinations and topical imiquimod therapy. The tumor measured 2 cm^3^. **(C)** Patient B, hematoxylin and eosin stain of recurrent cervical cancer showing areas of invasive focally keratinizing, moderately differentiated squamous carcinoma involving entire thickness of the stroma (×20 magnification, scale 100 µm). **(D)** Patient B, tumor identified and biopsied 3–4 weeks after completion of chemo-radiation for stage 3B squamous cell cancer of the cervix, pelvic exam, PET/CT, and biopsies confirmed radiation-refractory, recurrent disease. **(E)** Radical hysterectomy and bilateral salpingo-oophorectomy following failed curative chemo-radiation (8,500 cGy total to point A) followed by two cycles of intratumoral vaccinations and imiquimod. No residual squamous cell carcinoma on pathological evaluation and negative HPV-HR testing of the cervix. Vaginal cuff ThinPrep™ 6 weeks post-operatively was negative for dysplasia/malignancy and HPV-HR DNA.

Following the first 2-week cycle of immune therapy, the ulcerated, granular tumor surface was now flat and smooth, the margins of the ulcer were initially irregular and asymmetric were now well-defined and symmetric. The odor resolved and a yellowish, purulent, exudate was present (Figure 1A). Following the second intratumoral vaccination cycle, patient A missed her 2-week follow-up appointment for her third immunotherapy cycle. Eleven weeks after her second cycle, patient A returned for the third vaccination cycle. Examination of the right groin tumor was now less than 2 cm^3^ (Figure 1B). The third intratumoral vaccination was administered along with fourteen nights of imiquimod. Unfortunately, this 90-year-old patient would not return to the clinic. Despite multiple phone conversations with her, she stated her tumor was gone and she did not want to travelled the 220-mile round-trip unless absolutely needed. The home hospice nurses documented complete resolution of the groin tumor 4 weeks following the third intratumoral cycle. The patient never required supplementary oxygen. Nine months after her first intratumoral injection, patient A died in her sleep at 91 years old. The death certificate, completed by hospice physician, reported the cause of death as “natural causes.”

### Patient B

The patient was a 41-year-old Caucasian female, diagnosed with stage IIIB, HPV-HR positive, moderately differentiated squamous cell carcinoma of the cervix (Figure 1C). The patient was treated with pelvic irradiation (4500 cGy) with concurrent cisplatin (40 mg/m^2^) followed by two low-dose-rate cesium brachytherapy implants (total 8500 cGy to Point A). By the second tandem and ovoid application, the cervical tumor had completely resolved. Four weeks after her second cesium application, Patient B presented to the office complaining of vaginal discharge. Exam found a 3–4 cm recurrent, grade 1, squamous cell carcinoma, (Figure 1D). PET/CT imaging documented an isolated central cervical cancer recurrence with no evidence of metastatic disease. The immunologic therapy as described (Table 1) was offered following Bioethics committee approval.

There are no curative therapies to treat radiation refractory cervix cancer unless an isolated central cervical recurrence is present without evidence of metastatic disease. Patient B met the criteria for pelvic exenterative surgery. The experimental immune therapy was started while preparing for surgery. The first cycle of intratumoral injections of HPV-L1 vaccine was given followed by intravaginal 0.2% imiquimod suppositories every night until the second intratumoral injection 14 days later. This patient’s tumor produced the same characteristics as patient A’s tumor at 14 days. The surface of Patient B’s cervical cancer was no longer granular, irregular, and friable as seen initially but had a smooth, symmetric tumor surface. As observed with patient A’s groin tumor 2 weeks after the first injection, Patient B’s tumor decreased less than 25% following the first cycle. Surgery was scheduled 8 days after the second injection. On the day of surgery, examination under anesthesia demonstrated complete resolution of her radiation refractory cervix cancer. Frozen sections on two cervical biopsies were negative for persistent cancer. Therefore, in light of these new findings, the planned pelvic exenteration was aborted and post-radiation, radical hysterectomy with upper vaginectomy, bilateral salpingo-oopherctomy and pelvic and para-aortic lymphadenectomy was performed (Figure 1E). Post-operative pathology confirmed all tissue including 23 lymph nodes, cervix, upper vagina, and uterus were negative for both malignancy and HPV-HR genotypes. Three months following surgery, patient’s exam, HPV-HR testing, ThinPrep™ pap, and repeat PET/CT were all negative for disease. The patient is currently living with no evidence of disease over 8 years following treatment with this experimental immune therapy.

## Discussion

This paper emphasizes the use of sequential, targeted vaccinations of the FDA-approved, quadrivalent, HPV-L1 antigens vaccine injected throughout the previously irradiated tumor. HPV-L1 peptide has been found to produce an effective anti-tumor response [1]. The vaccination technique used for these patients was developed to treat women with HPV-related lower genital tract intra-epithelial neoplasia. Imiquimod, an FDA-approved Toll-like receptor 7 (TLR7) agonist, has been successfully used as a vaccine adjuvant demonstrating anti-tumor immune responses with radiation therapy and vaccines and improves the recognition and uptake of both DNA viruses and radiation induced tumor-associated antigens (neoantigens) [2, 3]. This TLR7-agonist facilitates antigen processing of tumor-specific antigens in regional lymph nodes [4]. This readily available topical medication improves several localized immune processes creating a TH1-dominant microenvironment within the tumor and most importantly, regional lymph nodes [5, 6].

There are several studies describing vaccine-based immunotherapies to treat malignant tumors. A complete review of therapeutic vaccines can be found elsewhere [7], most demonstrate cellular immune responses [3, 7, 8, 9], but few therapeutic vaccines studies report complete, durable responses as described in these two patients with radiation-refractory squamous cell malignancies. This report describes the first account in which the combination of fractionated radiation, sequential intratumoral vaccination and topical imiquimod therapy was used to produced complete, durable responses in two patients with radiation refractory cancers. A recent case report (2018) describes the successful use of systemic and intratumoral vaccinations of the recent, FDA-approved, nonavalent, HPV-L1 vaccine (Gardasil 9 ^TM^) treating 90-year-old women with multiple cutaneous, basaloid, squamous cell skin cancers [10]. Though this patient was not treated with nor failed radiation therapy, the authors reported similar tumor changes at 2 weeks post-vaccination as we observed at 2 weeks in our cases. Additionally, no specific side-effects or complications occurred [10].

**FIGURE 2:**
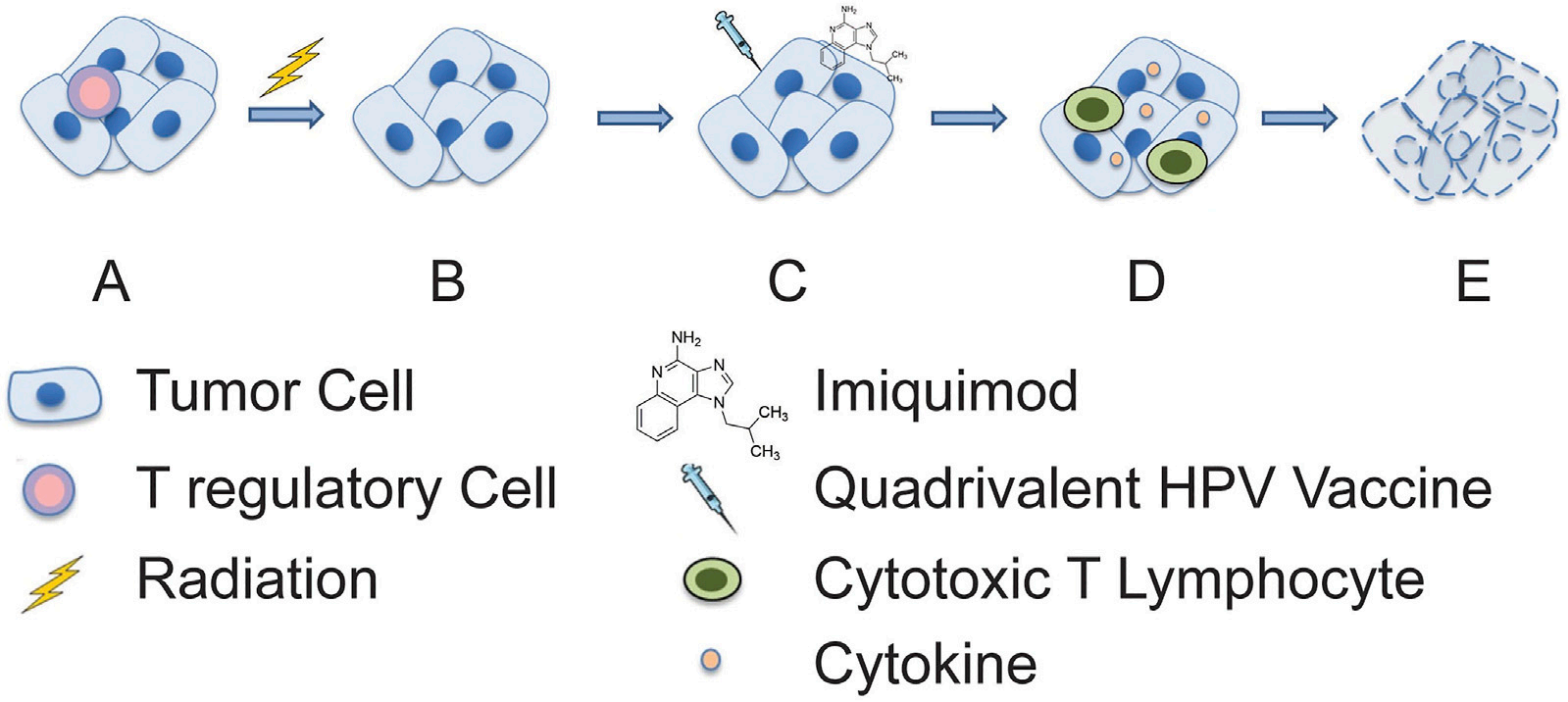
Proposed mechanism of synergistic anti-tumor activity of radiation and combination immunotherapy. **(A)** An immunosuppressive tumor microenvironment is initially present, and **(B)** subsequently an immune permissive microenvironment is created through depletion of immunosuppressive leukocytes (e.g., regulatory T cells) by radiotherapy. This is then followed by **(C)** treatment with imiquimod and quadrivalent HPV vaccine which **(D)** stimulates cytokine production and adaptive immune responses, respectively. **(E)** This enables synergistic tumor cell killing and resolution of cancer.

Radiation therapy is vital in changing the tumor’s well-established, immunosuppressive microenvironment [3]. Fractionated radiation has been reported to produces several tumor-specific biologic and phenotypic cellular changes making tumor cells more sensitive to immunogenic cell death. Some of the important changes include the upregulation of tumor cell’s MHC class 1 and 2 antigens and death receptors such as Fas/CD95 [11]. We postulate that the innate immune system’s nonspecific and rapid response. It may have occurred with the first cycle of intratumoral vaccinations in the form of macrophages, NK and dendritic cells [12, 13]. The cytotoxic Th1 anti-tumor effects are augmented by radiation-induced production of neoantigens and the rapid induction of tumor lysis. This leads to the release, uptake, and processing of neoantigens followed by transport to regional lymph nodes. Importantly, the radiation-induced neoantigens are only present on cancer cells [14, 15]. These tumor-specific antigens allow immune targeting and cross-presentation facilitating the recognition of the radiated tumors heterogeneous mutations producing a sort of personalized “*in-situ* vaccine.” [3, 12, 14] There is the possibility that the HPV-L1 vaccine which was injected throughout the tumor to account for genetic heterogeneity may have had an effect in attracting local macrophage, NK, and dendritic cells (Figure 2). Moreover, we cannot completely exclude the contribution of the trauma from repeated “needling” of the tumor, which may have added to the inflammatory responses.

Radiation therapy, when use as a single treatment modality, has not consistently produced therapeutic responses outside of the radiated field known as “abscopal effects” without the addition of adjuvant immunotherapies [3, 12]. Unfortunately, patient A refused post-treatment chest imaging which could have objectively documented an “abscopal response” of her pulmonary metastases. The fact she lived 9 months without requiring supplemental oxygen, beginning 2 weeks after her first intratumoral vaccination other, clinical evidence of, at least, a partial-abscopal response.

From a safety standpoint, this immune protocol produced no discernible toxicities in either patient. Our observations suggest that combination of imiquimod and quadrivalent HPV vaccine is likely less toxic and more tolerable than systemic cytotoxic chemotherapies. Most immune therapies do not result in cross-resistance with conventional therapies [16, 17]. Therefore, the possibility of using them in combination or sequentially with other standard therapies could be an option.

## Conclusion

We describe two female patients presenting with radiation-refractory, HPV-associated, squamous cell carcinomas of the lower genital tract facing death, patient A, or pelvic exenteration, patient B. Each patient was treated with an experimental therapy consisting of intratumoral injections of the preventative, quadrivalent HPV-L1 vaccine and topical application of imiquimod within 4 weeks following radiation therapy. Each patient demonstrated complete resolution of the treated tumor in addition to patient A’s clinical abscopal response of her pulmonary metastases allowing her to live an additional 9 months 2 weeks after starting this combination immune therapy. Currently, Patient B is alive with no evidence of disease 8 years following the modification of her surgery to a less radical operation due to resolution of her central recurrence 8 days after her second cycle of intratumoral therapy. We believe radiation therapy followed by intratumoral vaccinations and topical TLR-7 agonist resulted in a “prime-boost” response facilitating an appropriate cytotoxic antitumor response. If this combination of cancer therapies can be validated in appropriate clinical trials; this therapy could offer an urgent and unmet need for patients with treatment-refractory, HPV-related malignancies. Additionally, both medications in this report are FDA-approved for other conditions and have established safety profiles. The combination of radiation therapy followed by sequential intratumoral vaccinations and topical imiquimod could conveniently translate to the clinic setting and offers potential cost savings.

## Data Availability

The original contributions presented in the study are included in the article/Supplementary Material, further inquiries can be directed to the corresponding author.
