# Supplementary material for: Case Report: Intra-Tumoral Vaccinations of Quadrivalent HPV-L1 Peptide Vaccine With Topical TLR-7 Agonist Following Recurrence: Complete Resolution of HPV-HR-Associated Gynecologic Squamous Cell Carcinomas in Two Patients
Source: Pathol Oncol Res. 2021 Dec 20;27:1609922. doi: 10.3389/pore.2021.1609922 (PMC8720759; doi:10.3389/pore.2021.1609922)
Supplement: Supplementary file 1 [file DataSheet1.docx]

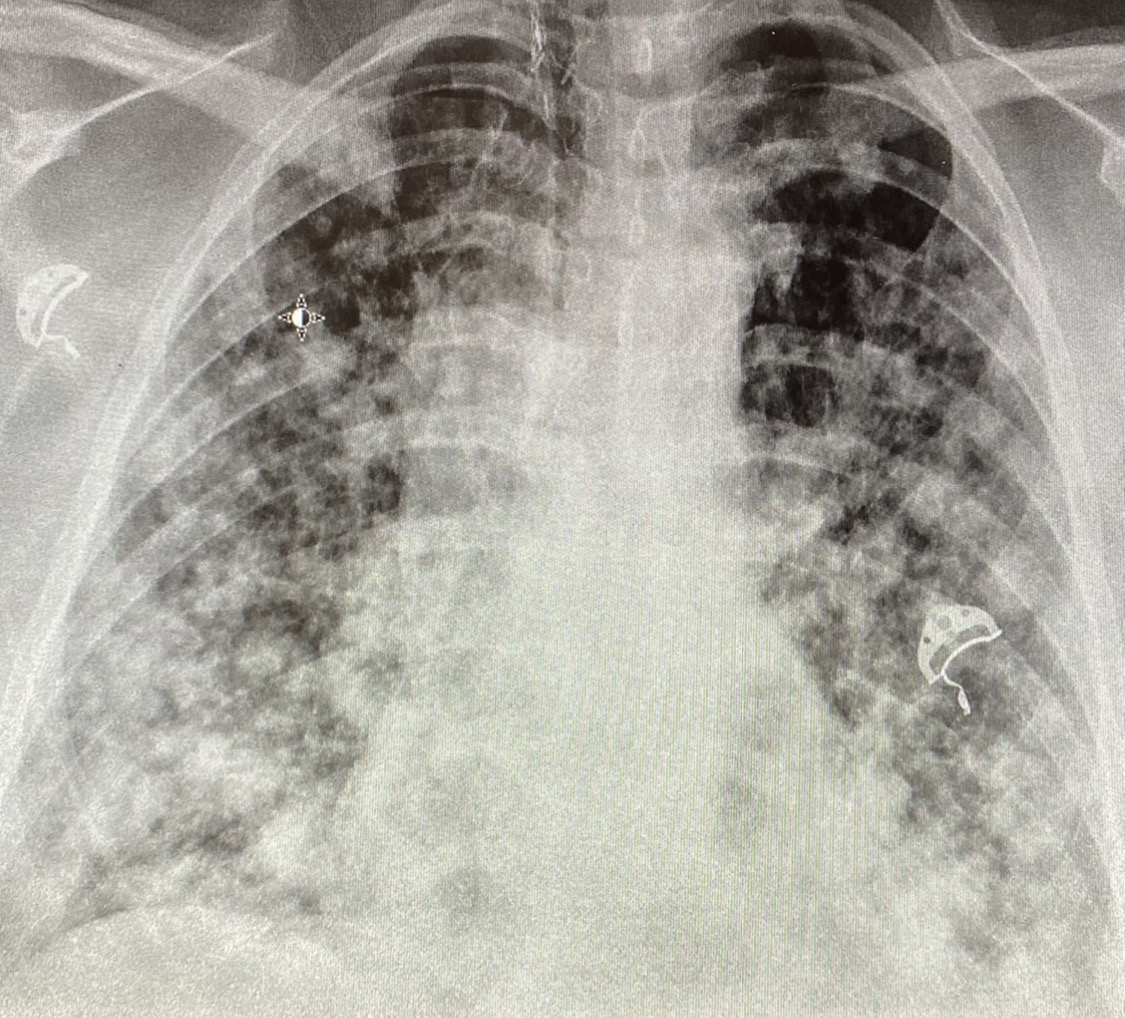


**Figure S1:** Patient A’s Chest x-ray showing bilateral pulmonary metastasis described as a “snow storm” pattern.
